# Effects of *Withania somnifera* (Ashwagandha) on Hematological and Biochemical Markers, Hormonal Behavior, and Oxidant Response in Healthy Adults: A Systematic Review

**DOI:** 10.1007/s13668-023-00481-0

**Published:** 2023-07-10

**Authors:** Adrián Gómez Afonso, Diego Fernandez-Lazaro, David P. Adams, Aniol Monserdà-Vilaró, Cesar I. Fernandez-Lazaro

**Affiliations:** 1 Faculty of Physical Activity Sport Sciences, European University of Madrid, 28670 Madrid, Spain; 2 Departamento de Biología Celular, Genética, Histología y Farmacología, Facultad de Ciencias de la Salud, Campus de Soria, Universidad de Valladolid, Soria, 42004 Spain; 3 Grupo de Investigación Reconocido “Neurobiología”, Facultad de Medicina, Universidad de Valladolid, Valladolid, 47005 Spain; 4 University of Galway, University Road, Galway, H91 TK33 Ireland; 5 Department of Preventive Medicine and Public Health, School of Medicine, University of Navarra, 31008 Pamplona, Spain; 6 IdiSNA, Navarra Institute for Health Research, 31008 Pamplona, Spain

**Keywords:** *Withania somnifera*, Ashwagandha, Plant supplement, Biomarkers, Healthy adults, Systematic review

## Abstract

**Purpose of Review:**

*Withania somnifera* (L.) Dunal *(Ws)* is a common herb plant that has been used for centuries to treat a wide range of conditions, particularly certain chronic diseases due to its antidiabetic, cardioprotective, antistress, and chondroprotective effects, among many others. No conclusive evidence, however, exists about the potential health effects of *Ws* in adults without chronic conditions. We aimed to evaluate the current evidence on the health benefits of *Ws* supplementation in healthy adults.

**Recent Findings:**

Based on the Preferred Reporting Items for Systematic Reviews and Meta-Analyses (PRISMA) guidelines, we systematically reviewed studies indexed in Web of Science, Scopus, and PubMed to assess the effects of *Ws* on hematological and biochemical markers, hormonal behavior, and oxidant response in healthy adults. Original articles published up to March 5, 2022, with a controlled trial design or pre-post intervention design, in which supplementation of *Ws* was compared to a control group or data prior to intervention were included. Among 2,421 records identified in the search, 10 studies met the inclusion criteria. Overall, most of the studies reported beneficial effects of the *Ws* supplementation, and no serious adverse events were reported. Participants supplemented with *Ws* displayed reduced levels of oxidative stress and inflammation, and counterbalanced hormone levels. No evidence of the beneficial effects of *Ws* supplementation on hematological markers was reported.

**Summary:**

*Ws* supplementation appears to be safe, may regulate hormone levels, and has potent anti-inflammatory and antioxidant effects. However, further studies are needed to elucidate the relevance of its application.

**Supplementary Information:**

The online version contains supplementary material available at 10.1007/s13668-023-00481-0.

## Introduction

*Withania somnifera* (L.) Dunal *(Ws)* is a small woody shrub that belongs to the Solanaceae family *(Solanaceae Juss.)*. It is commonly found in the drier regions of western India and is known variously as “Ashwagandha,” “Indian winter cherry”, or “poison gooseberry”. The plant is considered one of the most important herbs in Ayurveda — the traditional system of medicine in India [1]. Moreover, Ashwagandha is often used in *Rasayana*, one of the disciplines of Ayurveda, which promotes longevity by delaying ageing and preventing disease [2, 3]. While Ashwagandha has been traditionally used for different purposes for centuries for its mental and physical health benefits [4], it is also considered a plant-dried nutritional component [5]. Therefore, Ashwagandha can be considered both an herbal medicine and a plant-dried nutritional component.

The therapeutic effects of Ashwagandha are primarily attributed to its bioactive compounds rather than its nutritional content alone [6]. This plant contains numerous bioactive compounds, including more than 12 alkaloids, 40 withanolides, and several sitoindosides that confer antimicrobial, anti-inflammatory, antitumoral, antistress, antidiabetic, cardiovascular, neuroprotective, and immunomodulatory activity [7]. Most of the pharmacological activity of Ashwagandha has been attributed to withaferin A and glycowithanoloides (sitoindosides VII–X). Whitanolides are steroidal lactones; their action and appearance resemble ginsenosides, the active compounds in Asian ginseng, namely *Panax ginseng* [6]. Ashwagandha is also considered an adaptogen that helps to restore homeostasis by counteracting external stimuli as non-specific regulators, via several mechanisms of action associated with the homeostatic preservation of the hypothalamic-pituitary-adrenal (HPA) axis and the regulation of key mediators of the stress response [8].

The popularity of *Ws* has extended to non-Asian regions such as the United States where sales have risen rapidly, particularly after the COVID-19 pandemic [9]. In 2020, Ashwagandha experienced the greatest sales increase in the US mainstream multi-outlet channel (grocery outlets, drug outlets, and selected retailers across mass merchandisers) by 185.2% respect to 2019, reaching over $31 million sales and ranking 12^th^ in the US herbal supplements retail channel [10]. Numerous studies have demonstrated the benefits of Ashwagandha on physical performance [11–16] as well as for individuals with medical conditions such as diabetes [17–19], cognitive dysfunction [20–22], infertility [23–25], and anxiety and stress [26, 27]. Recent systematic reviews have supported such findings [28, 29•, 30, 31, 32•] corroborated the effects of *Ws* on strength and power, cardiorespiratory fitness, and fatigue and recovery, while the systematic review conducted by Durg et al. [29•] demonstrated improvements in blood glucose (post-prandial blood glucose and HbAlc) levels and lipid profile (total cholesterol, triglycerides, low-density lipoprotein [LDL], very low-density lipoprotein [VLD], and high-density lipoprotein [HDL] levels) in diabetic patients. Other systematic reviews have determined the benefits of *Ws* on cognitive effects (executive function, attention, and cognitive tasks, reaction time) [30]; male infertility such as semen parameters (volume, concentration, and motility), hormonal profile (luteinizing hormone, testosterone, and prolactin), oxidative biomarkers (lipid peroxides, superoxide dismutase [SOD], catalase, and glutathione) and antioxidant vitamins in seminal plasma (vitamin A, C, and E) [31]; and anxiety and stress (related questionnaires) [32•].

A number of studies have also assessed the positive effects of *Ws* among general population, i.e. healthy adults without chronic conditions or healthy adults with mild stress resulting in inconsistent findings related to health biomarkers [20, 33–35]. While *Ws* seems to improve hormonal response [33], particularly in stressed individuals [20], no hematological improvements of *Ws* were observed [33–35]. To date, no systematic review has been conducted to evaluate the potential health benefits of Ashwagandha on healthy adults. Therefore, we aimed to systematically review current evidence on the effects of *Ws* on health biomarkers and determine whether the supplementation of *Ws* improves hematological and biochemical biomarkers, hormonal behavior, and oxidant response in healthy adults, including those with moderate stress.

We framed the research question of the study using the population, intervention, comparison, and outcome (PICO) model according to the evidence-based medicine (EBM) [36] as follows: *Population*: healthy adults (without any chronic condition); *Intervention*: supplementation with *Ws* (alone); *Comparison*: placebo/control group or pre/post comparison data group; *Outcomes*: hematological (hemoglobin [Hb] and hematocrit [HCT]); hormonal (levels of testosterone, dehydroepiandrosterone [DHEA], and cortisol); antioxidant (malondialdehyde [MDA], SOD, and total antioxidant capacity [TAC]); biochemical (creatinine [Cr], creatine kinase [CK], aspartate aminotransferase [AST], alanine aminotransferase [ALT], alkaline phosphatase [ALP], and C-reactive protein [CRP]) markers. These biomarkers were included as outcomes as they are commonly investigated in studies of health markers and medicinal plant research.

## Methods

### Search Strategy

This systematic review focused on the following research question: “Does *Ws* supplementation have any health benefit for healthy adults without chronic conditions?” We developed a structured search using the databases Medline (PubMed), Web of Science (WOS), and Scopus for articles published from database inception to March 5, 2022, restricted to English language articles, and based on the PRISMA (Preferred Reporting Items for Systematic Reviews and Meta-Analyses) guidelines [37].

The search strategy included terms related to *Ws* and the outcomes as well as a combination of them with Medical Subject Headings (MeSH) index and Boolean operators: (“Ashwagandha” OR “*Withania somnifera*” OR “KSM66” OR “Shoden” OR “Indian Ginseng” OR “Poison Gooseberry” OR “Winter Cherry”) AND (“biological” OR “biochemical” OR “hematological” OR “hormonal” OR “enzymatic OR “inflammatory” OR “oxidant” OR “hemoglobin” OR “hematocrit” OR “testosterone” OR “dehydroepiandrosterone” OR “cortisol” OR “malondialdehyde” OR “superoxide dismutase” OR “total antioxidant capacity” OR “creatinine” OR “creatine kinase” OR” aspartate aminotransferase” OR “alanine aminotransferase” OR “alkaline phosphatase” OR “C-reactive protein”) AND (“adaptations” OR “markers” OR “effects” OR “analysis” OR “biomarkers” OR “indicators” OR “activity” OR “pathways”). Two reviewers (A.G. and A.M.) independently screened titles and abstracts. Full texts were sourced for relevant articles. Inclusion criteria were independently assessed for these two reviewers, and a third reviewer (C.I.F-L.) resolved any disagreements between them. Additional records were gleaned by conducting a ‘snowball’ search checking the reference lists of publications eligible for full-text review and using ResearchGate to identify potential articles not included in the aforementioned databases used in the study.

### Selection Criteria

We based selection of records on the following criteria: a) healthy adults without any chronic condition (including individuals with moderate levels of stress and excluding animal and/or in vitro studies); b) studies that assessed the effects of Ashwagandha supplementation alone (excluding multi-herbal formulas and any combination with other supplements); c) clinical trials, randomized and nonrandomized trials, and pre-test/post-test design studies (excluding editorials, reviews, notes, and any other non-original studies); d) studies that evaluated as outcomes (primary, secondary, or safety) any of the hematological, hormonal, antioxidant, and biochemical markers previously described; and e) studies with clear information on dosage and duration of *Ws* supplementation. We excluded all records that did not meet these criteria.

### Quality Assessment

We used the modified critical review form for quantitative studies developed by the McMaster University Occupational Therapy Evidence-Based Practice Research Group as critical appraisal tool [38]. This guide is appropriate for evaluation of randomized and non-randomized studies, as it is a comprehensive and reliable tool for assessing the methodological quality of quantitative evidence. It also suggests a threshold indicating quality appraisal.

The modified McMaster appraisal tool includes 16 items (Supplemental Table s1). For each item that an article meets, 1 point is assigned, otherwise, 0 points are assigned. The quality assessment was categorized as “poor” (≤ 8 points), “fair” (9–10 points), “good” (11–12 points), “very good” (13–14 points), and “excellent” (≥ 15 points).

### Data Extraction

Two reviewers (A.G. and A.M.) examined and synthesized data from all selected studies into a comprehensive table using a standardized data extraction. A third reviewer (C.I.F-L.) resolved all inter-reviewer disagreements. Information extracted from the selected studies included name of the first author; publication year; country where the study was conducted; study design; sample size; participants’ sex and age; dosage, i.e., specific amount, number, frequency, and percentage of withanolides; timing of the supplementation; duration of intervention; outcomes; and results.

## Results

### Study Selection

**Fig. 1:**
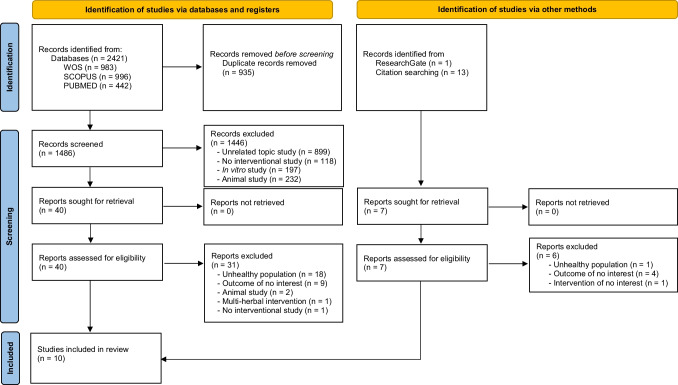
Flow diagram depicting the identification and selection processes of relevant studies according to Preferred Reporting Items for Systematic Reviews and Meta-Analyses (PRISMA) guidelines

The literature search resulted in 2,421 records. Among them, 1,486 were initially obtained from Medline, WOS, and Scopus, and 14 came from additional sources, e.g., ResearchGate and reference lists from relevant studies. After the exclusion of 935 duplicates, we screened a total of 1,486 articles identified from databases. Once title and abstract were evaluated, we considered 40 articles as potential records. After full-text review and evaluation the potential records coming from databases as well as other sources, 10 studies [15, 16, 20, 33–35, 39–42] were included in the systematic review (Fig. 1).

### Quality Assessment

Six studies [15, 16, 20, 39–41] were assessed as being of “excellent quality”; 3 studies [33, 35, 42] as having “very good quality”; and 1 study [34] as having “fair quality”. All studies met the minimum quality criteria (Table 1).

**Table 1:** Results of the methodological quality assessment of included studies — McMaster Critical Review Form for Quantitative Studies [38]

**Study**	**Item**																**Total**	**%**	**Quality score**
	**1**	**2**	**3**	**4**	**5**	**6**	**7**	**8**	**9**	**10**	**11**	**12**	**13**	**14**	**15**	**16**			
Gopukumar et al. [20], 2021	1	1	1	1	1	1	1	1	1	1	1	1	1	1	1	1	16	100	E
Kuchewar et al. [33], 2014	1	1	1	1	1	1	1	1	1	0	1	1	1	1	0	1	14	87.5	VG
Lopresti et al. [39]., 2019	1	1	1	1	1	1	1	1	1	1	1	1	1	1	1	1	16	100	E
Lopresti et al. [40], 2019	1	1	1	1	1	1	1	1	1	1	1	1	1	1	1	1	16	100	E
Pingali et al. [42], 2013	1	1	1	1	1	0	1	1	1	1	1	1	1	1	1	0	14	87.5	VG
Raut et al. [34], 2012	0	1	0	0	1	0	1	1	1	1	1	1	0	1	1	0	10	62.5	F
Tiwari et al. [41], 2021	1	1	1	1	1	1	1	1	1	1	1	1	1	1	1	1	16	100	E
Verma et al. [35], 2021	1	1	1	1	0	1	0	1	1	1	1	1	1	1	1	0	13	86.7	VG
Wankhede et al. [15], 2015	1	1	1	1	1	0	1	1	1	1	1	1	1	1	1	1	15	93.8	E
Ziegenfuss et al. [16], 2018	1	1	1	1	1	0	1	1	1	1	1	1	1	1	1	1	15	93.8	E

Item 1: study purpose; item 2: literature review; item 3: study design; item: 4 blinding; item 5: sample description; item 6: sample size; item 7: ethics and consent; item 8: validity of outcomes; item 9: reliability of outcomes; item 10: intervention description; item 11: statistical significance; item 12: statistical analysis; item 13: clinical importance; item 14: conclusions; item 15: clinical implications; item 16: study limitations

*0* not fulfilled criterion, *1* fulfilled criterion, *E* excellent, *VG* very good, *G* good, *F* fair

### Characteristics of the Participants and Interventions

The number of total participants at baseline was 542 (380 men and 162 women). All the participants were healthy individuals without any chronic conditions. One study [39] included participants who were overweight/obese (mean body mass was 27.9 kg/m^2^ and 26.7 kg/m^2^ for intervention and control groups, respectively) with low-to-moderate anxiety levels (score ranged between 6–17 points in the Hamilton Anxiety Rating Scale). Another study [40] included healthy participants with light-to-moderate anxiety levels (Profile of Mood States Fatigue-Inertia score > 50^th^ percentile, or POMs total score or Vigor-Activity score < 50^th^ percentile); and 1 study [20] included adults with moderate level of stress (score ranged between 14–24 points in the 10-item Perceived Stress Scale). In 3 studies [15, 16, 41] participants reported being physically active, having some experience with resistance training [15], being recreationally active [16], and athletic [41] (Table 2).

**Table 2:** Characteristics of participants and supplementation protocols of the selected studies

**Characteristics**	**Types**	**Study**
Participants	Healthy	[33–35, 42]
	Healthy physically active	[15, 16, 41]
	Healthy with light-to-moderate anxiety levels	[20, 40]
	Healthy overweight/obese with mild/moderate symptoms of fatigue	[39]
Supplementation product	Manufactured	[15, 20, 33, 35, 39–41]
	Registered product^®^	[16, 42]
	No reported	[34]
% Withanolides of the supplementation product	No less than 5%	[15, 20, 35, 41]
	No less than 10%	[16, 42]
	35%	[39, 40]
	No reported	[33, 34]
Total dose (mg)	240	[40]
	300	[20]
	500	[16]
	600	[15, 35, 39, 41]
	750–1000-1250	[34]
	1000	[33, 42]
Duration	2 weeks	[42]
	30 days	[34]
	8 weeks	[15, 35, 39, 41]
	60 days	[40]
	90 days/12 weeks	[16, 20]
	6 months	[33]
Dose schedule	a.m	[16]
	After breakfast	[20]
	After dinner	[39, 40]
	a.m. and p.m	[15, 33, 34]
	No reported	[35, 41, 42]

*a.m.* ante meridiem, *p.m.* post meridiem, *mg* milligrams

Intervention protocols varied by dose, duration, and schedule. Doses of *Ws* supplementation*,* varied from 240 mg [40] to 1,250 mg [34], with 600 mg as the most common dose used [15, 35, 39, 41]. Supplementation duration ranged from 2 weeks [42] to 6 months [33]. Most studies utilized an 8-week period [15, 35, 39, 41]. Investigators administered supplementation in the morning [16], after breakfast [20], after dinner [39, 40], and in the morning and in the evening [15, 33, 34] (Table 2). Overall, subjects tolerated Ashwagandha supplementation well. In 1 study [34], however, a subject reported unusual increases in appetite and libido as well as hallucinogenic effects with vertigo on the third day of supplementation.

### Outcome Evaluation

Table 3 summarizes the information of the studies included in the present review.

**Table 3:** Studies included in the systematic review of the effect of *Withania somnifera* on hematological and biochemical markers, hormonal behavior, and oxidant response in healthy adults

**First author, year of publication, and country**	**Study design**	**Participants *(*baseline sample size and characteristics, withdrawals, and final group sample size)**	**Intervention**	**Outcomes**	**Results**
Gopukumar et al. [20], 2021, India	A randomized, double-blind, placebo-controlled study	70 ♂ & 60 ♀ healthy with moderate levels of stress^a^ Age (range): 20–55 y 5 withdrawals/lost to follow-up 2 participants CG 3 participants IG	1 × 300 mg sustained-released *Ws* After breakfast 90 days	Cortisol ALT AST	IG *vs* CG ↓ Cortisol Change from baseline ↓Cortisol ALT (no abnormal changes) AST (no abnormal changes) Cr (no abnormal changes)
Kuchewar et al. [33], 2014, India	Randomized, double-blind, placebo-controlled trial	10 ♂ & 20 ♀ Age (range): 18–45 y No withdrawals reported 9 participants CG 11 participants IG	2 × 500 mg *Ws* a.m. and p.m. intakes 6 months	Hb SOD MDA	Change from baseline ↔ Hb ↑ SOD ↓MDA
Lopresti et al. [39], 2019, Australia	Randomized, double-blind, placebo-controlled, crossover trial	57 ♂ healthy overweight/obese with mild/moderate symptoms of fatigue^b^ Age (mean ± SD): CG to IG: 51.7 ± 1.2 y IG to CG: 50.1 ± 1.3 y 14 withdrawals/lost to follow-up 43 participants completed the study 23 from CG to IG 20 from IG to CG	2 × 300 mg *Ws* (35% withanolides) 2 h separate from meals, preferably after dinner 8 wk with crossover period	Testosterone DHEA Cortisol	IG *vs* CG ↑Testosterone ↑ DHEA ↔ Cortisol
Lopresti et al. [40], 2019, Australia	Randomized, double-blind, placebo-controlled trial	37 ♂ & 23 ♀ with low-to-moderate anxiety^3^ Age (mean ± SD): CG: 40.2 ± 2.4 y IG: 42.2 ± 2.4 y No withdrawals/lost to follow-up reported 30 participants CG 30 participants IG	1 × 240 mg *Ws* (35% withanolides) After dinner 60 days	Testosterone DHEA Cortisol	IG *vs* CG ↔ Testosterone ↓DHEA ↓Cortisol IG *vs* CG for men ↔ Testosterone ↓DHEA ↔ Cortisol IG *vs* CG for women ↔ Testosterone ↔ DHEA ↔ Cortisol IG: change from baseline ↑Testosterone ↓DHEA ↓Cortisol IG: change from baseline for men ↑ Testosterone ↔ DHEA ↓Cortisol IG: change from baseline for women ↔ Testosterone ↔ DHEA ↓Cortisol
Pingali et al. [42], 2013, India	Randomized, double-blind, placebo controlled, crossover trial	20 ♂ healthy Age (mean ± SD): 25.1 ± 2.3 y No withdrawals reported	2 × 2 pillsx250 mg *Ws* (at least 10% of withanolides) 2 wk with crossover period	Cortisol MDA CRP	IG *vs* CG ↓Cortisol ↓ MDA ↓ CRP Change from baseline ↓Cortisol ↓ MDA ↓ CRP
Raut et al. [34], 2012, India	Prospective, open- label trial	12 ♂ & 6 ♀ with no training program in the preceding last month Age (mean ± SD): 24.3 ± 2.1 y No withdrawals/lost to follow-up reported	1–10 day: 750 mg *Ws* 11–20 day: 1000 mg *Ws* 21–30 day: 1250 mg *Ws* a.m. and p.m. intakes 30 days	Cr ALT AST ALP	Change from baseline ↑ Cr ↔ ALT ↔ AST ↔ ALP
Tiwari et al. [41], 2021, India	Prospective, randomized, double-blind, placebo-controlled study	37 ♂& 13 ♀ athletic participants Age (mean ± SD): CG: 28.8 ± 7.5 y IG: 29.3 ± 8.8 y No withdrawals/lost to follow-up reported 25 participants CG 25 participants IG	2 × 300 mg (> 5% withanolides) 8 wk	TAC	IG *vs* CG ↑ TAC Change from baseline ↑ TAC
Verma et al. [35], 2021, India	Randomized, double-blind, placebo-controlled trial	40 ♂ & 40 ♀ healthy Age (mean ± SD): CG: 29.22 ± 7.51 y IG: 31.80 ± 8.91 y No withdrawals/lost to follow-up reported	2 × 300 mg *Ws* (> 5% withanolides) 8 wk	Hb ALT AST ALP	Change from baseline ↔ Hb ↔ ALT ↔ AST ↔ ALP
Wankhede et al. [15], 2015, India	Prospective, randomized, double-blind, placebo-controlled study	57 ♂ with little experience in resistance training Age (mean ± SD): CG: 29 ± 9 y IG: 28 ± 8 y 7 withdrawals/lost to follow-up reported 50 participants completed the study 25 participants CG 25 participants IG	2 × 300 mg *Ws* (5% withanolides) After awaking and before going to bed 8 wk AND resistance training program 3 days per wk	Testosterone CK	Change from baseline ↑Testosterone ↓CK IG *vs* CG change from baseline ↑Testosterone ↓ CK
Ziegenfuss et al. [16], 2018, United States	Randomized, double-blind, placebo-controlled trial	40 ♂ recreationally active Age (mean ± SD): CG: 28.6 ± 7.6 y IG: 24.4 ± 4.2 y 2 withdrawals/lost to follow-up 38 participants completed the study 19 participants CG 19 participants IG	500 mg *Ws* (10% withanolides) a.m. intake 12 wk AND resistance training program 4 days per wk	Hb HCT Cr ALT AST ALP	IG *vs* CG ↓ Hb ↓HCT ↔ Cr ↔ ALT ↔ AST ↔ ALP Change from baseline ↓ Hb ↔ HCT ↑ Cr ↔ ALT ↔ AST ↔ ALP

↑ = significant increase; ↓ = significant decrease; ↔ = no significant change

*ALP* alkaline phosphatase, *ALT* alanine aminotransferase, *AST* aspartate aminotransferase, *CG* control group, *Cr* creatinine, *ESR* erythrocyte sedimentation rate, *HB* Hemoglobin, *HCT* Hematocrit, *IG* intervention group, *MDA* Malondialdehyde, *SD* Standard Deviation, *SOD* superoxide dismutase, *TAC* Total Antioxidant Capacity, *wk* weeks, Ws *Withania somnifera*

^a^The Perceived Stress Scale 10-item score ranged from 14 to 24 (moderate level of stress)

^b^Profile of Mood States [POMS] Fatigue-Inertia score > 50^th^ percentile, or POMs total score or Vigor- Activity score < 50^th^ percentile

^c^Hamilton Anxiety Rating Scale score ranged between 6 and 17 (low to moderate anxiety severity)

#### Hematological Markers

Three studies [16, 33, 35] evaluated the effects of *Ws* on hematological markers. Hb was the most frequently studied hematological marker in the selected studies. Ziegenfuss et al. [16] observed a significant reduction in Hb and HCT levels when compared *Ws* supplementation group with the control group. None of the other studies found significant changes in Hb [33, 35].

#### Hormonal Response

Testosterone levels were examined in 3 studies [15, 39, 40]. Two studies reported significant increases in testosterone levels in the Ashwagandha intervention group relative to the control [15, 39]. Examining changes from baseline, 2 studies [15, 40] observed significant increases in testosterone levels. In a study conducted by Lopresti et al. [40]﻿﻿, however, increases in testosterone levels did not occur in females. Regarding DHEA levels, 2 studies showed contradictory findings when compared *Ws* supplementation group with a control group [39, 40]. Four studies investigated cortisol levels [20, 39, 40, 42], observing significantly decreased levels from baseline to the end of the intervention and also between intervention and control groups [20, 40, 42].

#### Oxidant Response

Several studies assessed the effects of *Ws* on antioxidant markers [33, 41, 42]. Two investigated MDA levels [33, 42], 1 evaluated SOD levels [33], and another examined TAC levels [42]. All reported significant changes in antioxidant markers [33, 41, 42]. MDA levels were significantly reduced among participants in the Ashwagandha intervention group compared to the control group [33, 42]. Investigators also observed MDA changes from baseline to the end of the study in the intervention group [42]. Moreover, intervention-group participants showed reduced SOD [33] and augmented TAC levels [41] relative to controls. Significant TAC changes from baseline were additionally observed in the intervention group [41].

#### Biochemical Markers

The effect of *Ws* on ALP, ALT, and AST were evaluated in 3 studies [16, 34, 35] observing no significant changes. In 2 of these studies [16, 34], researchers additionally observed augmented Cr levels changes from baseline to the end of the intervention. Nonetheless, no significant changes were reported between the intervention and control groups [16]. Another study [20] reported no abnormal changes in AST, ALT, and Cr, but results were not reported on statistical significance. Investigators also assessed CK and CRP levels and found significantly reduced changes in the Ashwagandha intervention group compared to the control [15, 42].

## Discussion

To the best of our knowledge, this is the first systematic review to critically evaluate the effects of *Ws* supplementation on hematological and biochemical markers, hormonal behavior, and antioxidant response in healthy adults with no medical chronic conditions. Ten studies met the pre-specified inclusion criteria. Overall, participants supplemented with *Ws* displayed significant improvements on hormonal response, reduced oxidative stress, and inflammation. There was no clear evidence of the beneficial effects of *Ws* supplementation on hematological markers.

### *Withania somnifera* supplementation

Supplementation doses administered in interventions ranged from 240 mg [40] to 1,250 mg [34], from 2 weeks [42] to 6 months [33] without reports of any serious adverse events. We were not able to identify a clear duration pattern to ensure that long-term supplementation affects outcomes differently. Moreover, researchers reported no substantial changes in ALP, ALT, and AST levels, markers frequently used to assess drug-induced liver toxicity [16, 20, 34, 35]. This finding is consistent with other human clinical study in which mild to moderate transient adverse events have been reported without any serious adverse events [43]. Murine studies have also reported the safety of Ashwagandha without mutagenicity or genotoxicity, [44••] or toxicity when rats received the maximum recommended *Ws* dosage [45]. In the United States, regulators consider *Ws* a dietary supplement [46], and its annual sales there have increased more than 180%, making it the 12^th^ top-selling herb in 2020 [10]. Furthermore, the World Anti-Doping Agency does not list Ashwagandha as a banned substance [47].

### Hematological Markers

Animal and human studies have reported significant increases in Hb levels [14, 48, 49]. None included in this systematic review, however, identified improvements in hematological markers during Ashwagandha supplementation [16, 33, 35]. Improvements in Hb levels were observed with Ashwagandha supplementation in mice with myelosuppression [48]. This activity may be explained because *Ws* counteracted myelosuppression drug toxicity and restored bone-marrow activity. Previous studies [50–53] have demonstrated anticancer, antitoxic, and antioxidant activities of Ashwagandha. Investigators have attributed these properties to its bioactive compounds such as Withaferin A and some alkaloids. The capability of *Ws* to scavenge reactive oxygen species (ROS), e.g., their selective induction in cancer cells, might be the underlying mechanism [54].

However, it appears that *Ws* does not directly affect erythropoietic activity in humans. Nonetheless, it may produce hematological effects by modulating oxidative stress that prevent changes in the erythrocyte membrane, avoid premature removal of the cell, and reduced red blood cell’s life span. The intensity of the oxidative stress modulation may be sufficient to improve hematological markers in animals or individuals exposed to physically demanding environments, such as horses [49] or athletes [14], but not in healthy humans [33, 35] or recreational subjects [16] whose ROS production is not particularly high. Such findings support the role of *Ws* as an adaptogen supplement in physically demanding situations.

### Hormonal Response

Investigators have reported beneficial effects of *Ws* supplementation on testosterone [15, 40] and DHEA levels [39]. These results are consistent with animal studies in which *Ws* was shown to increase gonadotropins and hormones, particularly testosterone [55]. Other human studies have demonstrated similar findings, reporting significantly higher levels of testosterone and luteinizing hormone in infertile men [56]. Nonetheless, in 1 study included in the present review, the levels of DHEA when supplementing with *Ws* decreased [40]. We hypothesized that the reduction of DHEA levels may be influenced by the elevated baseline cortisol levels of the participants (participants with low-to-moderate anxiety). DHEA is an endogenous precursor of testosterone produced by the adrenal cortex [57] and is interconnected with cortisol with opposing functions, having DHEA attenuating effects on cortisol levels [58, 59]. The modulatory effects of Ashwagandha on cortisol levels in individuals with stress/anxiety have been reported previously [27, 60] and are consistent with 3 studies included in this review [20, 40, 42].

Investigators have also suggested that Ashwagandha may have mitigating effects on the HPA axis activity via cortisol-levels reduction. Specifically, the effect of *Ws* on Gamma-Aminobutyric Acid (GABA) and serotonergic pathways may result in a reduction of the HPA axis activation, diminishing the secretion of corticotropin-releasing hormone (from the hypothalamus) and the adrenocorticotropic hormone (from the pituitary), leading to reduced levels of cortisol released from the adrenal cortex [61]. These findings seem to occur in populations with high or moderate levels of cortisol [20, 27, 40, 42, 60], but are unclear in populations with low cortisol [39]. Alternatively, *Ws* supplementation may also modulate key-enzyme activity in physiological pathways associated with testosterone production, leading to increased levels in individuals with low cortisol levels [15, 39, 56] but not those with high cortisol levels [40]. The anti-inflammatory and antioxidant properties of *Ws* may also influence the effects on testosterone levels because of their multidirectional activities [62–64]. These results support the use of Ashwagandha as an adaptogen to counterbalance and equilibrate biomarker levels to create hormonal level balances.

### Oxidant Response

The 3 studies [33, 41, 42] that examined the effects of *Ws* supplementation on antioxidant activity reported significant improvements in MDA [33, 42], SOD [33], and TAC levels [42]. These results are consistent with previous animal studies, in vitro and in vivo analyses, and clinical trials in healthy athletic individuals [41, 64–66]. Moreover, investigators have reported beneficial effects of *Ws* in individuals with conditions linked to oxidative stress [43].

As noted above, investigators have postulated Withaferin A — a highly oxygenated lactone — and some of its alkaloids such as the sitoindosides VII-X, as powerful antioxidants [50–52]. High concentrations of phenolic, flavonoids, and antioxidant activity have been characterized in *Ws* by high-performance liquid chromatography (HPLC) [67]. These compounds allow *Ws* to counterbalance ROS generation as well as reverse changes in lipid peroxidation and damaged cells. ROS bind with cell membranes and generate toxic lipid peroxidatives such as MDA [53].

Withaferin A is also a potent inducer of nuclear factor erythroid 2-related factor 2 (Nrf2), a transcription factor that regulates the expression of antioxidant enzyme genes involved in the response to oxidative stress through activation of the PTEN/PI3K/Akt pathway, exerting a cytoprotective role [68, 69]. Nrf2 activation creates downstream production of antioxidant enzymes such as catalase and SOD, glutathione (GSH) synthase, GSH peroxidase, GSH reductase, thioredoxin (Trx) and Trx reductase, and antioxidant proteins such as heat shock protein 70 (HSP70) and Heme oxygenase-1 (HO-1) [70]. Unlike vitamins and coenzymes, these enzymatic and protein antioxidants are not consumed in the redox reaction, providing a longer duration of action because they no longer required continuous production [71]. The antioxidant properties of the bioactive compounds of *Ws* in healthy individuals support its use to improve oxidative balance status and may prevent oxidative stress-associated diseases.

### Biochemical Markers

This review examined Cr [16, 34], CK [15], transaminases (ALP, ALT, and AST) [16, 34, 35], and CRP [42] as biochemical markers. In these studies, *Ws* supplementation demonstrated a reduction in CK [15] and CRP activity [42], potent inflammatory markers. Consistent evidence has demonstrated several anti-inflammatory properties of *Ws* on inflammation-mediated chronic diseases [51], specifically systemic lupus erythematosus [72], inflammatory bowel disease [73], rheumatoid arthritis [74], and coronavirus disease (COVID-19) [75•] as a potential agent to attenuate the cytokine storm. Researchers have attributed most of the anti-inflammatory properties of *Ws* to withanolides, primarily Withaferin A [76, 77]. Although the mechanisms responsible for the anti-inflammatory effects of withanolides are not fully understood, *Ws* may act by interacting with mediators of the inflammatory cell signaling pathway such as NF-κB, signaling kinases, HSP90, Nrf2, and the inflammasome complex.

The family of nuclear factor NF-kappa-B (NF-κB) transcription factors are involved in several inflammation-driven chronic diseases, making NF-κB a therapeutic target for individuals with elevated NF-κB levels. In this context, *Ws* can interfere with the NF-κB pathway and mediate its inhibition [76•]. *Ws* relies on the development of potent protein kinase inhibitor activity. *Ws* can block protein kinases signaling cascades that play a central role in inflammatory pathways [51, 76•] Moreover, the kinase inhibition seems to occur during the inhibition of nitric oxide production, having a positive impact on the inflammation process. The downregulation and destabilization of the activity HSP involved in important regulatory kinase pathways, appear to be another mechanism to explain the anti-inflammatory action of *Ws*. As discussed above, Ashwagandha can modulate oxidative stress by regulating Nrf2 [68, 69]. As oxidative stress often occurs at inflammatory sites, apparently triggering chronic inflammation [78], Nrf2 activation may also explain how *Ws* reduces inflammation [78]. Lastly, *Ws* may reduce inflammation by inhibiting some inflammasomes, cytokines, and other multiprotein proinflammatory proteins [76•]. Although in this review did not evaluate mediators of the inflammatory cell signaling pathways, the reduction in CK and CRP levels found in the selected studies may suggest anti-inflammatory effects of *Ws*, even in individuals with low levels of inflammation. This supports the use of Ashwagandha to prevent chronic inflammatory diseases.

Regarding the results on ALP, ALT, and ASP, participants in the studies included in this review did not significantly change their levels after Ashwagandha supplementation [16, 34, 35] or not experience abnormal changes [20]. Cr levels, however, increased from baseline, but these changes were not observed when compared to control groups [16, 34]. On one hand, these findings suggest the safety of *Ws* supplementation as no hepatic damage or toxicity was indicated. As such, investigators have attributed additional nephroprotective and hepatoprotective effects to Ashwagandha in toxic-induced elevations in AST, ALT, bilirubin, urea, and Cr levels in rats [79, 80].

Alternatively, the increased levels of Cr that Raut et al. [34] and Ziegenfuss et al. [16] observed were less likely to be related to renal failure as the serum concentration of Cr remained within the normal range (0.9 to 1.3 mg/dL for adult males and 0.6 to 1.1 mg/dL for adult females) and were most likely due to muscle mass increments caused by *Ws* [81]*.* The increased effect of Ashwagandha on testosterone and the decreased effect on the levels of cortisol may have contributed to muscle growth, that consequently, may result in increased Cr levels. Moreover, in the study conducted by Ziegenfuss et al. [16] the intervention group included a training program that may additionally influence Cr levels via changes in muscle mass.

The authors of this review acknowledge some limitations. First, a limited number of manuscripts met the inclusion criteria. Second, the considerable heterogenicity of the studies regarding outcomes, supplementation dosage, and the length of intervention, did not allow us to conduct a meta-analysis. The great variability in *Ws* supplementation merits caution when interpreting results; nevertheless, there is robust evidence of the health benefits of *Ws* in populations with chronic conditions [29, 30, 51, 60]. The variability in the protocols observed in this systematic review warrants future developments on the design of control trials aimed to determine appropriate dose, percentage of withanolides, and duration of *Ws* supplementation to observe its potential health benefits. The results of more homogeneous studies may be integrated in future meta-analyses and contribute to the evidence on this field. Moreover, the limited number of existing studies in the literature conducted among healthy population without chronic conditions calls for further research on this population to corroborate the findings.

Despite the aforementioned limitations, strengths of our systematic review lie in the use of the PRISMA guidelines [37],﻿ reliance of three main databases and grey literature to conduct the search, as well as the use of the modified McMaster appraisal tool for methodological quality assessment [38] to ensure all selected records met the minimum-quality criteria.

## Conclusions

This is the first systematic to assess the potential health benefits of *Ws* supplementation in adults without chronic conditions. The evidence presented in this systematic review showed that *Ws* supplementation is safe. Given improvements in certain biomarkers, it may also benefit healthy individuals. Although *Ws* may act as an adaptogen to counterbalance and adjust some physiological markers outside the normal range, e.g., hematological and hormonal, it may exert a more potent anti-inflammatory and antioxidant effect, even in low inflammatory and oxidant status, that could further be utilized to prevent chronic inflammatory conditions. Further research, however, is needed to confirm the potential benefits on health of *Ws* supplementation in healthy adults without chronic diseases.

## Supplementary Information

Below is the link to the electronic supplementary material.

Supplementary file1 (DOCX 23 kb)

## Data Availability

Data are available upon reasonable request to the corresponding author.

## References

[1] Mukherjee PK, Harwansh RK, Bahadur S, Banerjee S, Kar A, Chanda J (2017). Development of Ayurveda – Tradition to trend. J Ethnopharmacol.

[2] Govindarajan R, Vijayakumar M, Pushpangadan P (2005). Antioxidant approach to disease management and the role of ‘Rasayana’ herbs of Ayurveda. J Ethnopharmacol.

[3] Goyal M (2018). Rasayana in perspective of the present scenario. Ayu.

[4] Singh N, Bhalla M, de Jager P, Gilca M (2011). An Overview on Ashwagandha: A Rasayana (Rejuvenator) of Ayurveda. Afr J Tradit Complement Altern.

[5] Krishnamurthy SR, Sarala P (2010). Proximate nutritive values and mineral components of *Withania somnifera* (Linn.) Dunal. J Chem.

[6] Mirjalili MH, Moyano E, Bonfill M, Cusido RM, Palazón J (2009). Steroidal lactones from *Withania somnifera*, an ancient plant for novel medicine. Molecules.

[7] Kulkarni SK, Dhir A (2008). *Withania somnifera*: an Indian ginseng. Prog Neuro-Psychopharmacol Biol Psychiatry.

[8] Panossian AG, Efferth T, Shikov AN, Pozharitskaya ON, Kuchta K, Mukherjee PK (2021). Evolution of the adaptogenic concept from traditional use to medical systems: Pharmacology of stress- and aging-related diseases. Med Res Rev.

[9] Council for Responsible Nutrition C. Dietary Supplement Usage Up Dramatically During Pandemic. In: CRN’s COVID-19 Survey on Dietary Supplements: Consumer Insights on Usage and Attitudes about Dietary Supplements in Light of the Coronavirus Pandemic. 2020. https://www.crnusa.org/COVID19survey. Accessed 20 Nov 2022.

[10] Smith T, Majid F, Eckl V, Reynolds CM. Herbal supplement sales in US increase by record-breaking 17.3% in 2020 - American Botanical Council. HerbalGram. 2021;52–65.

[11] Choudhary B, Shetty A, Langade DG (2015). Efficacy of Ashwagandha (*Withania somnifera* [L.] Dunal) in improving cardiorespiratory endurance in healthy athletic adults. Ayu.

[12] Shenoy S, Chaskar U, Sandhu JS, Paadhi MM (2012). Effects of eight-week supplementation of Ashwagandha on cardiorespiratory endurance in elite Indian cyclists. J Ayurveda Integr Med.

[13] Tripathi R, Salve B, Petare A, Raut A, Rege N. Effect of Withania somnifera on physical and cardiovascular performance induced by physical stress in healthy human volunteers. Int J Basic Clin Pharmacol. 2016;5:2510–6. 10.18203/2319-2003.ijbcp20164114.

[14] Malik A, Mehta V, Dahiya V (2013). Effect of Ashwagandha (Withania Somnifera) root powder supplementation on the Vo2 max. and hemoglobin in hockey players. Int J Behav Soc Mov Sci..

[15] Wankhede S, Langade D, Joshi K, Sinha SR, Bhattacharyya S (2015). Examining the effect of Withania somnifera supplementation on muscle strength and recovery: A randomized controlled trial. J Int Soc Sports Nutr.

[16] Ziegenfuss T, Kedia A, Sandrock J, Raub B, Kerksick C, Lopez H (2018). Effects of an aqueous extract of *Withania somnifera* on strength training adaptations and recovery: the STAR trial. Nutrients.

[17] Nayak S, Nayak S, Panda BK, Das S (2015). A Clinical Study on management of stress in type-2 diabetes mellitus (Madhumeha) with ashwagandha (Withania Somnifera). Ayushdhara.

[18] Andallu B, Radhika B (2000). Hypoglycemic, diuretic and hypocholesterolemic effect of Winter cherry (*Withania somnifera*, Dunal) root. Indian J Exp Biol.

[19] Usharani P, Fatima N, Kumar CU, Kishan PV (2015). Evaluation of a highly standardized *Withania somnifera* extract on endothelial dysfunction and biomarkers of oxidative stress in patients with type 2 diabetes mellitus: A randomized, double blind, placebo controlled study. Int J Ayurveda Pharma Res.

[20] Gopukumar K, Thanawala S, Somepalli V, Rao TSS, Thamatam VB, Chauhan S (2021). Efficacy and safety of ashwagandha root extract on cognitive functions in healthy, stressed adults: a randomized, double-blind, placebo-controlled study. Evid Based Complement Alternat Med.

[21] Chengappa KNR, Bowie CR, Schlicht PJ, Fleet D, Brar JS, Jindal R (2013). Randomized placebo-controlled adjunctive study of an extract of withania somnifera for cognitive dysfunction in bipolar disorder. J Clin Psychiatry.

[22] Pingali U, Pilli R, Fatima N (2013). Effect of standardized aqueous extract of Withania somnifera on tests of cognitive and psychomotor performance in healthy human participants. Pharmacognosy Res.

[23] Gupta A, Mahdi AA, Shukla KK, Ahmad MK, Bansal N, Sankhwar P (2013). Efficacy of *Withania somnifera* on seminal plasma metabolites of infertile males: A proton NMR study at 800 MHz. J Ethnopharmacol.

[24] Mahdi AA, Shukla KK, Ahmad MK, Rajender S, Shankhwar SN, Singh V (2009). Withania somnifera improves semen quality in stress-related male fertility. Evid Based Complement Altern Med..

[25] Ahmad MK, Mahdi AA, Shukla KK, Islam N, Rajender S, Madhukar D (2010). Withania somnifera improves semen quality by regulating reproductive hormone levels and oxidative stress in seminal plasma of infertile males. Fertil Steril.

[26] Langade D, Thakare V, Kanchi S, Kelgane S (2021). Clinical evaluation of the pharmacological impact of ashwagandha root extract on sleep in healthy volunteers and insomnia patients: A double-blind, randomized, parallel-group, placebo-controlled study. J Ethnopharmacol.

[27] Salve J, Pate S, Debnath K, Langade D (2019). Adaptogenic and anxiolytic effects of ashwagandha root extract in healthy adults: a double-blind, randomized, placebo-controlled clinical study. Cureus..

[28] Bonilla DA, Moreno Y, Gho C, Petro JL, Odriozola-Martínez A, Kreider RB (2021). Effects of Ashwagandha (*Withania somnifera*) on physical performance: systematic review and bayesian meta-analysis. J Funct Morphol Kinesiol.

[29] Durg S, Bavage S, Shivaram SB (2020). Withania somnifera (Indian ginseng) in diabetes mellitus: a systematic review and meta-analysis of scientific evidence from experimental research to clinical application. Phyther Res.

[30] Ng QX, Loke W, Foo NX, Tan WJ, Chan HW, Lim DY (2020). A systematic review of the clinical use of Withania somnifera (Ashwagandha) to ameliorate cognitive dysfunction. Phyther Res.

[31] Durg S, Shivaram SB, Bavage S (2018). Withania somnifera (Indian ginseng) in male infertility: An evidence-based systematic review and meta-analysis. Phytomedicine.

[32] Akhgarjand C, Asoudeh F, Bagheri A, Kalantar Z, Vahabi Z, Shab-bidar S (2022). Does Ashwagandha supplementation have a beneficial effect on the management of anxiety and stress? A systematic review and meta-analysis of randomized controlled trials. Phytother Res.

[33] Kuchewar VV, Borkar MA, Nisargandha MA (2014). Evaluation of antioxidant potential of Rasayana drugs in healthy human volunteers. Ayu.

[34] Raut AA, Rege NN, Tadvi FM, Solanki PV, Kene KR, Shirolkar SG (2012). Exploratory study to evaluate tolerability, safety, and activity of Ashwagandha (*Withania somnifera*) in healthy volunteers. J Ayurveda Integr Med.

[35] Verma N, Gupta SK, Tiwari S, Mishra AK (2021). Safety of Ashwagandha root extract: a randomized, placebo-controlled, study in healthy volunteers. Complement Ther Med.

[36] Sackett DL, Richardson WS, Rosenberg W, Haynes RB (1997). Evidence Based Medicine.

[37] Page MJ, Moher D, Bossuyt PM, Boutron I, Hoffmann TC, Mulrow CD (2021). PRISMA 2020 explanation and elaboration: updated guidance and exemplars for reporting systematic reviews. BMJ.

[38] Law M, Stewart D, Letts L, Pollock N, Bosch J, Westmorland M. Guidelines for critical review of qualitative studies. McMaster Univ Occup Ther evidence-based Pract Res Gr. 1998.

[39] Lopresti AL, Drummond PD, Smith SJ (2019). a randomized, double-blind, placebo-controlled, crossover study examining the hormonal and vitality effects of Ashwagandha (*Withania somnifera*) in aging, overweight males. Am J Mens Health.

[40] Lopresti AL, Smith SJ, Malvi H, Kodgule R, Wane D (2019). An investigation into the stress-relieving and pharmacological actions of an ashwagandha (*Withania somnifera*) extract: a randomized, double-blind, placebo-controlled study. Med.

[41] Tiwari S, Gupta SK, Pathak AK (2021). A double-blind, randomized, placebo-controlled trial on the effect of Ashwagandha (*Withania somnifera* dunal.) root extract in improving cardiorespiratory endurance and recovery in healthy athletic adults. J Ethnopharmacol.

[42] Pingali U, Pilli R, Fatima N, Packer PL (2013). Effect of *Withania somnifera* extract on mental stress induced changes in hemodynamic properties and arterial wave reflections in healthy subjects. Curr Top Nutraceutical Res.

[43] Lopresti AL, Smith SJ (2021). Ashwagandha (*Withania somnifera*) for the treatment and enhancement of mental and physical conditions: a systematic review of human trials. J Herb Med..

[44] •• Tandon N, Yadav SS. Safety and clinical effectiveness of *Withania somnifera* (Linn.) Dunal root in human ailments. J Ethnopharmacol. 2020;255:112768. 10.1016/J.JEP.2020.112768. **This manuscript summarizes the evidence of the clinical effectiveness and safety of Ws in pre-clinical and clinical trials.** 10.1016/j.jep.2020.112768 32201301

[45] Antony B, Benny M, Kuruvilla BT, Kumar Gupta N, Sebastian A, Jacob S. Acute and sub chronic toxicity studies of purified Withania somnifera extract in rats. Int J Pharm Pharm Sci. 2018;10:41–6. 10.22159/IJPPS.2018V10I12.29493.

[46] National Institutes of Health (NIH). U.S. Food and Drug Administration. In: Diet. Suppl. Fact Sheets. https://ods.od.nih.gov/factsheets/list-all/. Accessed 2 Dec 2022.

[47] World Anti-Doping Agency (WADA). The Prohibited List. In: Athletes & Support Personnel. 2022. https://www.wada-ama.org/en/prohibited-list. Accessed 3 Dec 2022.

[48] Ziauddin M, Phansalkar N, Patki P, Diwanay S, Patwardhan B (1996). Studies on the immunomodulatory effects of Ashwagandha. J Ethnopharmacol.

[49] Priyanka G, Anil Kumar B, Lakshman M, Manvitha V, Kala KB (2020). Adaptogenic and immunomodulatory activity of Ashwagandha root extract: an experimental study in an equine model. Front Vet Sci.

[50] Dutta R, Khalil R, Green R, Mohapatra SS, Mohapatra S (2019). *Withania Somnifera* (Ashwagandha) and Withaferin A: potential in integrative oncology. Int J Mol Sci.

[51] White PT, Subramanian C, Motiwala HF, Cohen MS (2016). Natural withanolides in the treatment of chronic diseases. Adv Exp Med Biol.

[52] Haque A, Shahriar M, Hossain MI, Sharmin FA, Akhter S, Haque MA (2013). InVitro antioxidant and free radical scavenging activity of *Withania Somnifera* root. Iosr J Pharm.

[53] Harikrishnan B, Subramanian P, Subash S (2008). Effect of Withania somnifera root powder on the levels of circulatory lipid peroxidation and liver marker enzymes in chronic hyperammonemia. E-J Chem.

[54] Chang HW, Li RN, Wang HR, Liu JR, Tang JY, Huang HW (2017). Withaferin A induces oxidative stress-mediated apoptosis and DNA damage in oral cancer cells. Front Physiol.

[55] Rahmati B, Moghaddam MHG, Khalili M, Enayati E, Maleki M, Rezaeei S. Effect of *Withania somnifera* (L.) Dunal on sex hormone and gonadotropin levels in addicted male rats. Int J Fertil Steril. 2016;10:239–44. 10.22074/IJFS.2016.4915. 10.22074/ijfs.2016.4915 PMC4948077 27441058

[56] Ambiye VR, Langade D, Dongre S, Aptikar P, Kulkarni M, Dongre A (2013). Clinical evaluation of the spermatogenic activity of the root extract of Ashwagandha (Withania somnifera) in oligospermic males: A pilot study. Evid Based Complement Alternat Med..

[57] Kroboth PD, Salek FS, Pittenger AL, Fabian TJ, Frye RF (1999). DHEA and DHEA-S: a review. J Clin Pharmacol.

[58] Buoso E, Lanni C, Molteni E, Rousset F, Corsini E, Racchi M (2011). Opposing effects of cortisol and dehydroepiandrosterone on the expression of the receptor for Activated C Kinase 1: implications in immunosenescence. Exp Gerontol.

[59] Pinto A, Malacrida B, Oieni J, Serafini MM, Davin A, Galbiati V (2015). DHEA modulates the effect of cortisol on RACK1 expression via interference with the splicing of the glucocorticoid receptor. Br J Pharmacol.

[60] Chandrasekhar K, Kapoor J, Anishetty S (2012). A prospective, randomized double-blind, placebo-controlled study of safety and efficacy of a high-concentration full-spectrum extract of Ashwagandha root in reducing stress and anxiety in adults. Indian J Psychol Med.

[61] Tafet GE, Nemeroff CB (2020). pharmacological treatment of anxiety disorders: the role of the HPA axis. Front Psychiatry.

[62] Mohamad NV, Wong SK, Wan Hasan WN, Jolly JJ, Nur-Farhana MF, Ima-Nirwana S (2019). The relationship between circulating testosterone and inflammatory cytokines in men. Aging Male.

[63] Shahraki MR, Noshahr ZS, Ahmadvand H, Nakhaie A (2016). Anti-nociceptive and anti-inflammatory effects of Withania somnifera root in fructose fed male rats. J Basic Clin Physiol Pharmacol.

[64] Noshahr ZS, Shahraki MR, Ahmadvand H, Nourabadi D, Nakhaei A (2015). Protective effects of Withania somnifera root on inflammatory markers and insulin resistance in fructose-fed rats. Rep Biochem Mol Biol.

[65] Pal A, Kumar KH, Bhushan B, Saharan V (2017). Ashwagandha root extract inhibits acetylcholine esterase, protein modification and ameliorates H_2_O_2_-induced oxidative stress in rat lymphocytes. Pharmacogn J.

[66] Bhattacharya SK, Satyan KS, Ghosal S (1997). Antioxidant activity of glycowithanolides from Withania somnifera. Indian J Exp Biol.

[67] Alam N, Hossain M, Khalil MI, Moniruzzaman M, Sulaiman SA, Gan SH (2011). High catechin concentrations detected in Withania somnifera (ashwagandha) by high performance liquid chromatography analysis. BMC Complement Altern Med.

[68] Yan Z, Guo R, Gan L, Lau WB, Cao X, Zhao J (2018). Withaferin A inhibits apoptosis via activated Akt-mediated inhibition of oxidative stress. Life Sci.

[69] Palliyaguru DL, Chartoumpekis DV, Wakabayashi N, Skoko JJ, Yagishita Y, Singh SV (2016). Withaferin A induces Nrf2-dependent protection against liver injury: Role of Keap1-independent mechanisms. Free Radic Biol Med.

[70] Bocci V, Valacchi G (2015). Nrf2 activation as target to implement therapeutic treatments. Front Chem.

[71] Cai J, Xu L, Tang H, Yang Q, Yi X, Fang Y (2014). The role of the PTEN/PI3K/Akt pathway on prognosis in epithelial ovarian cancer: a meta-analysis. Oncologist.

[72] Minhas U, Minz R, Bhatnagar A (2011). Prophylactic effect of Withania somnifera on inflammation in a non-autoimmune prone murine model of lupus. Drug Discov Ther.

[73] Pawar P, Gilda S, Sharma S, Jagtap S, Paradkar A, Mahadik K (2011). Rectal gel application of Withania somnifera root extract expounds anti-inflammatory and muco-restorative activity in TNBS-induced Inflammatory Bowel Disease. BMC Complement Altern Med.

[74] Kumar G, Srivastava A, Sharma SK, Rao TD, Gupta YK (2015). Efficacy & safety evaluation of Ayurvedic treatment (Ashwagandha powder & Sidh Makardhwaj) in rheumatoid arthritis patients: a pilot prospective study. Indian J Med Res.

[75] • Saggam A, Limgaokar K, Borse S, Chavan-Gautam P, Dixit S, Tillu G, et al. *Withania somnifera* (L.) Dunal: opportunity for clinical repurposing in COVID-19 management. Front Pharmacol. 2021;12:623795. 10.3389/fphar.2021.623795. **This narrative review discusses the potential utility of the use of Ws in the clinical managements of COVID-19.** 10.3389/fphar.2021.623795 PMC8126694 34012390

[76] • Logie E, Vanden Berghe W. Tackling chronic inflammation with withanolide phytochemicals-A withaferin A perspective. Antioxidants. 2020;9:1107. 10.3390/antiox9111107 . **In-depth approach of the anti-inflammatory mechanisms of action of Ws to restore immune homeostasis.** 10.3390/antiox9111107 PMC7696210 33182809

[77] Sun GY, Li R, Cui J, Hannink M, Gu Z, Fritsche KL, et al. *Withania somnifera* and its withanolides attenuate oxidative and inflammatory responses and up-regulate antioxidant responses in BV-2 microglial cells. NeuroMolecular Med. 2016;18:241–52. Available from: 10.1007/s12017-016-8411-0. 10.1007/s12017-016-8411-0 27209361

[78] Biswas SK (2016). Does the interdependence between oxidative stress and inflammation explain the antioxidant paradox?. Oxid Med Cell Longev.

[79] Jeyanthi T, Subramanian P (2009). nephroprotective effect of *Withania somnifera*: a dose-dependent study. Ren Fail.

[80] Sultana N, Choudhury Shimmi S, Tanveer M, Parash H, Akhtar J (2012). Effects of Ashwagandha (*Withania somnifera*) root extract on some serum liver marker enzymes (AST, ALT) in gentamicin intoxicated rats. J Bangladesh Soc Physiol.

[81] Venkataraghavan S, Seshadri C, Sundaresan T, Revathi R, Rajagopalan V, Janaki K (1980). The comparative effect of milk fortified with ashwagandha and punarnava in children – a double blind study. J Res Ayur Sid..

