# Supplementary material for: Effects of Withania somnifera (Ashwagandha) on Hematological and Biochemical Markers, Hormonal Behavior, and Oxidant Response in Healthy Adults: A Systematic Review
Source: Curr Nutr Rep. 2023 Jul 10;12(3):465–77. doi: 10.1007/s13668-023-00481-0 (PMC10444651; doi:10.1007/s13668-023-00481-0)
Supplement: Supplementary file 1 — Supplementary file1 (DOCX 23 kb) [file 13668_2023_481_MOESM1_ESM.docx]

**Supplemental Table s1.** Criteria for the Modified McMaster Critical Review Form for Quantitative Studies.

| **[1. Purpose]** | |  |  |  | **[8. Validity of outcomes]** | |  |  |  |
| --- | --- | --- | --- | --- | --- | --- | --- | --- | --- |
|  | *Do the authors clearly state that the aim of the study?* | | | |  | *Did the authors use outcome measures that are considered to be valid for use in target populations to assess all outcome variables that are relevant to this review?* | | | |
|  |  |  |  |  |  |  |  |  |  |
|  | Yes | 1 |  |  |  |  |  |  |  |
|  | No or Unable to determine | 0 |  |  |  |  |  |  |  |
|  |  |  |  |  |  | Yes | 1 |  |  |
| **[2. Literature review]** | |  |  |  |  | No or Unable to determine | 0 |  |  |
|  | *Do the authors justify, by identifying gaps in the literature, the need to undertake further research into the study topic?* | | | |  |  |  |  |  |
|  |  |  |  |  | **[9. Reliability of outcomes]** | |  |  |  |
|  |  |  |  |  |  | *Did the authors use outcome measures whose reliability has been established for use in target populations to assess all outcome variables that are relevant to this review?* | | | |
|  | Yes | 1 |  |  |  |  |  |  |  |
|  | No or Unable to determine | 0 |  |  |  |  |  |  |  |
|  |  |  |  |  |  |  |  |  |  |
| **[3. Study design]** | |  |  |  |  | Yes | 1 |  |  |
|  | *Have the authors used a randomized controlled trial to answer study aims, that is, to investigate the study topic?* | | | |  | No or Unable to determine | 0 |  |  |
|  |  |  |  |  |  |  |  |  |  |
|  |  |  |  |  | **[10. Intervention description]** | |  |  |  |
|  | Yes | 1 |  |  |  | *Did the authors provide sufficient information to enable reproduction of the intervention?* | | | |
|  | No or Unable to determine | 0 |  |  |  |  |  |  |  |
|  |  |  |  |  |  | Yes | 1 |  |  |
| **[4. Blinding]** | |  |  |  |  | No or Unable to determine | 0 |  |  |
|  | *Have the authors used assessor blinding to minimize bias?* | | | |  |  |  |  |  |
|  |  |  |  |  | **[11. Statistical significance]** | |  |  |  |
|  | Yes | 1 |  |  |  | *Did the authors report the results for at least one outcome measure in line with study aim and in terms of statistical significance?* | | | |
|  | No or Unable to determine | 0 |  |  |  |  |  |  |  |
|  |  |  |  |  |  |  |  |  |  |
| **[5. Sample description]** | |  |  |  |  | Yes | 1 |  |  |
|  | *Have the authors described the sample in terms of age, gender, and at least one measure of symptom condition?* | | | |  | No or Unable to determine | 0 |  |  |
|  |  |  |  |  |  |  |  |  |  |
|  |  |  |  |  | **[12. Statistical analysis]** | |  |  |  |
|  | Yes | 1 |  |  |  | *Did the authors use appropriate statistical analyses in evaluating results according to their aim?* | | | |
|  | No or Unable to determine | 0 |  |  |  |  |  |  |  |
|  |  |  |  |  |  |  |  |  |  |
| **[6. Sample size]** | |  |  |  |  | Yes | 1 |  |  |
|  | *Have the authors justified their sample size through a power calculation or post hoc analysis (and recruited sufficient numbers)?* | | | |  | No or Unable to determine | 0 |  |  |
|  |  |  |  |  |  |  |  |  |  |
|  |  |  |  |  | **[13. Clinical importance]** | |  |  |  |
|  | Yes | 1 |  |  |  | *Did the authors reflect on the clinical importance of results for investigated populations in discussions?* | | | |
|  | No or Unable to determine | 0 |  |  |  |  |  |  |  |
|  |  |  |  |  |  |  |  |  |  |
| **[7. Ethics and consent]** | |  |  |  |  | Yes | 1 |  |  |
|  | *Have the authors documented ethical approval for the research and gained informed consent by participants?* | | | |  | No or Unable to determine | 0 |  |  |
|  |  |  |  |  |  |  |  |  |  |
|  |  |  |  |  | **[14. Conclusions]** | |  |  |  |
|  | Yes | 1 |  |  |  | *Did the authors provide appropriate conclusions considering the study method and results?* | | | |
|  | No or Unable to determine | 0 |  |  |  |  |  |  |  |
|  |  |  |  |  |  | Yes | 1 |  |  |
|  |  |  |  |  |  | No or Unable to determine | 0 |  |  |
|  |  |  |  |  |  |  |  |  |  |
|  | |  |  |  |  |  |  |  |  |
| **[15. Clinical implications]** | |  |  |  |  |  |  |  |  |
|  | *Did the authors discuss clinical implications of the results and in directing further research?* | | | |  |  |  |  |  |
|  |  |  |  |  |  |  |  |  |  |
|  | Yes | 1 |  |  |  |  |  |  |  |
|  | No or Unable to determine | 0 |  |  |  |  |  |  |  |
|  |  |  |  |  |  |  |  |  |  |
| **[16. Study limitations]** | |  |  |  |  |  |  |  |  |
|  | *Did the authors identify limitations of the study methodology and results?* | | | |  |  |  |  |  |
|  |  |  |  |  |  |  |  |  |  |
|  | Yes | 1 |  |  |  |  |  |  |  |
|  | No or Unable to determine | 0 |  |  |  |  |  |  |  |

Modifications: 1) essential criteria was limited from ‘all outcome variables’ to ‘all relevant variables to this systematic review’ in the ‘Validity of outcome’ and ‘Reliability of outcomes’ (itmes 8 and 9, respectively); and 2) essential criteria in the ‘Validity of outcome’ (item 8) was soften from ‘Did the authors use outcome measures that are valid…’ to ‘Did the authors use outcome measures that are considered valid…’; 3) an expression of ‘in discussions’ was added in essential criteria in the ‘Clinical importance’ (item 13); and 4) a score of ‘Unable to determine’ (score = 0) was added. Each item was given a score of either 0 (Did not fulfil criteria or Unable to determine) or 1 (fulfilled criteria completely).
